# Association Between Osseointegration of Lower Extremity Amputation and Mortality Among Adults

**DOI:** 10.1001/jamanetworkopen.2022.35074

**Published:** 2022-10-13

**Authors:** Jason Shih Hoellwarth, Kevin Tetsworth, Atiya Oomatia, Muhammad Adeel Akhtar, Haikun Xu, Munjed Al Muderis

**Affiliations:** 1 Limb Salvage and Amputation Reconstruction Center, Hospital for Special Surgery, New York, New York; 2 Department of Orthopaedic Surgery, Royal Brisbane and Women’s Hospital, Queensland, Australia; 3 Limb Reconstruction Centre, Macquarie University Hospital, Macquarie University, Macquarie Park, Australia; 4 Trauma and Orthopaedic Department, Victoria Hospital Kirkcaldy, NHS (National Health Service) Fife, Kirkcaldy, Scotland; 5 Inter-American Tropical Tuna Commission, La Jolla, California

## Abstract

**Importance:**

Transcutaneous osseointegration post amputation (TOPA) creates a direct linkage between residual bone and an external prosthetic limb, providing superior mobility and quality of life compared with a socket prosthesis. The causes and potential risks of mortality after TOPA have not been investigated.

**Objective:**

To investigate the association between TOPA and mortality and assess the potential risk factors.

**Design, Setting, and Participants:**

This observational cohort study included all patients with amputation of a lower extremity who underwent TOPA between November 1, 2010, and October 31, 2021, at a specialty orthopedic practice and tertiary referral hospital in a major urban center. Patients lived on several continents and were followed up as long as 10 years.

**Exposures:**

Transcutaneous osseointegration post amputation, consisting of a permanent intramedullary implant passed transcutaneously through a stoma and connected to an external prosthetic limb.

**Main Outcomes and Measures:**

Death due to any cause. The hypotheses tested—that patient variables (sex, age, level of amputation, postosseointegration infection, and amputation etiology) may be associated with subsequent mortality—were formulated after initial data collection identifying which patients had died.

**Results:**

A total of 485 patients were included in the analysis (345 men [71.1%] and 140 women [28.9%]), with a mean (SD) age at osseointegration of 49.1 (14.6) years among living patients or 61.2 (12.4) years among patients who had died. Nineteen patients (3.9%) died a mean (SD) of 2.2 (1.7) years (range, 58 days to 5 years) after osseointegration, including 17 (3.5%) who died of causes unrelated to osseointegration (most commonly cardiac issues) and 2 (0.4%) who died of direct osseointegration-related complications (infectious complications), of which 1 (0.2%) was coclassified as a preexisting health problem exacerbated by osseointegration (myocardial infarction after subsequent surgery to manage infection). No deaths occurred intraoperatively or during inpatient recuperation or acute recovery after index osseointegration (eg, cardiopulmonary events). Kaplan-Meier survival analysis with log-rank comparison and Cox proportional hazards regression modeling identified increased age (hazard ratio, 1.06 [95% CI, 1.02-1.09]) and vascular (odds ratio [OR], 4.73 [95% CI, 1.35-16.56]) or infectious (OR, 3.87 [95% CI, 1.31-11.40]) amputation etiology as risk factors. Notable factors not associated with mortality risk included postosseointegration infection and male sex.

**Conclusions and Relevance:**

These findings suggest that patients who have undergone TOPA rarely die of problems associated with the procedure but instead usually die of unrelated causes.

## Key Points

**Question:**

What are the causes and potential factors associated with death after lower extremity osseointegration?

**Findings:**

In this cohort study of 485 patients who underwent transcutaneous osseointegration post amputation and were followed up for as long as 10 years, 19 patients (4%) died, including 17 who died of causes unrelated to osseointegration and 2 who died of osseointegration-related causes. Risk factors for mortality included increased age at osseointegration and the indication for amputation being either for vascular or infectious etiology; factors not associated with mortality risk included postosseointegration infection and sex.

**Meaning:**

These findings suggest that patients considering osseointegration to improve their mobility and quality of life have a minimal risk of mortality.

## Teaser

This cohort study evaluates the incidence, causes, and factors associated with mortality among patients who have undergone transcutaneous osseointegration for amputation.

## Introduction

Transcutaneous osseointegration post amputation (TOPA),^1^ a procedure that creates a direct linkage between residual bone and an external prosthetic limb, continues to gain momentum as a viable rehabilitation alternative to traditional compression and suction prostheses. Osseointegration provides improved mobility and quality of life compared with traditional prostheses for most patients with lower extremity amputation.^2,3^ As with any major elective orthopedic surgical procedure, there are inherent risks to balance against the potential benefits,^4,5^ with 1 potential risk being associated mortality. It is therefore imperative to investigate this risk so that clinicians may provide better information and patients may make better-informed decisions regarding this method of surgical reconstruction.

A 2018 systematic review of osseointegration complications^6^ found that mortality has never been the focus of investigation. Our own searches of subsequent literature identified no studies that focused on mortality after osseointegration. Deaths reported in literature evaluating other aspects of osseointegration are rare, perhaps owing to careful patient selection that prioritizes healthy patients,^7,8^ relatively short follow-up, or underreporting given that death was never the primary outcome of investigation. It is important to understand to what extent patient mortality may be associated with preoperatively modifiable risk factors or avoidable or unavoidable postoperative complications or whether mortality may be completely unrelated to this surgical procedure. To address this knowledge gap, we evaluated the incidence, causes of, and factors potentially associated with mortality among patients who have undergone TOPA.

## Methods

The Macquarie University ethical committee approved this cohort study and waived the need for informed consent for record review based on the combination of 3 situations: first, the patients of specific interest were deceased; second, the only expected potential harm to living patients being a breach of confidentiality; and third, the number of patients to consent. The study followed the Strengthening the Reporting of Observational Studies in Epidemiology (STROBE) reporting guideline.

### Study Design

**Figure 1.**
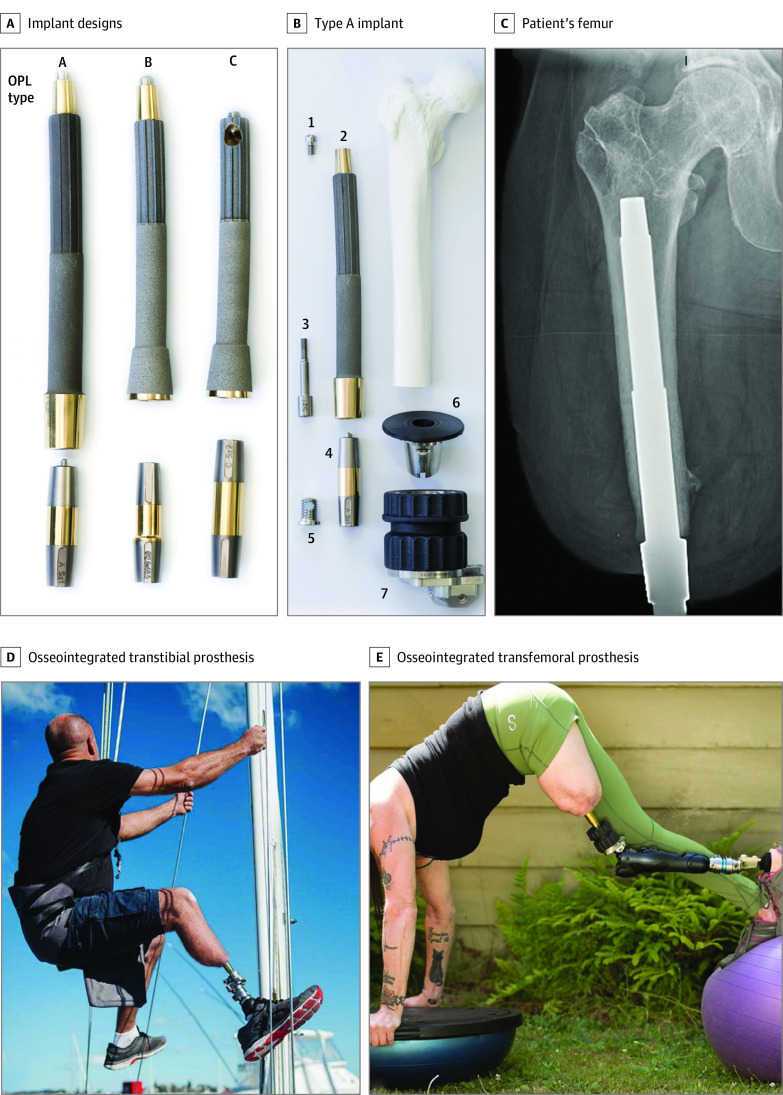
Presentation of Transcutaneous Osseointegration Post Amputation (TOPA) A, Photograph of common osseointegrated prosthetic limb (OPL [Permedica]) implant designs. The top portion is made of titanium with a textured intramedullary portion and smooth transcutaneous portion and is implanted into the patient’s residual limb. The dual cone below the implant is a connector to the external prosthetic limb. B, Exploded view of a type A implant with the components arranged at approximately the proximal-distal levels in which they would be once assembled and implanted in a patient who had undergone a femoral amputation. 1, Proximal cap screw; 2, OPL body; 3, safety screw; 4, dual cone transcutaneous adapter; 5, prosthesis adapter screw; 6, proximal connector; and 7, prosthetic connector. C, Anterior-posterior radiograph of a TOPA implant in a patient’s femur. The textured portion is intramedullary; the smooth portion is transcutaneous. D and E, Examples of activities that a patient with an osseointegrated transtibial (D) or transfemoral (E) implant can achieve with a skeletally connected prosthesis.

All patients in our prospectively maintained osseointegration registry were included in the evaluation. Patients from several continents had index osseointegration between November 1, 2010, and October 31, 2021, and were followed up for as long as 10 years. Indication criteria for patients considering osseointegration surgery have been described previously^8,9^ and are summarized as follows. Generally, patients considered for osseointegration (Figure 1) are skeletally mature adults who (1) report pain or mobility dissatisfaction with their traditional compression and suction prostheses; (2) have an intact limb with incapacitating pain, complex deformity, or profound distal weakness whose functional capacity is considered likely to be improved by amputation; or (3) have recently undergone amputation and prefer osseointegration to traditional socket prosthesis rehabilitation. The only situations we consider particularly rigid contraindications to osseointegration are modifiable compromises to successful bone and/or wound healing, such as active infection, malignant disease, or uncontrolled diabetes (although most patients are suitable for TOPA after stable completion of appropriate treatment). Patients whose transfemoral or transtibial osseointegration was performed at our primary institution were included in the study. Patients were excluded if their osseointegration was performed for the upper extremity or pelvis or if it was performed at outreach locations. This process yielded 485 patients.

Medical record and registry review were performed to identify patients who had died due to any cause. The patient registry is continuously updated, with attempts made to contact each patient at least annually for in-person or telemedicine-based clinical, radiographic, and research follow-up. For patients for whom the interval since their most recent review was longer than 1 year, we attempted to contact them or their family; if unsuccessful, we performed searches in Google and on social media platforms (Facebook, Instagram, Tik Tok, and Twitter). Of 485 patients, 398 (82.1%) were contacted within the prior 1 year, 455 (93.8%) were contacted within the prior 2 years, and 30 (6.2%) were contacted more than 2 years before this study. The circumstances leading to death were ascertained either by medical record review or from information provided by family members. All authors jointly discussed how to categorize each patient’s cause of death: (1) unrelated to osseointegration, (2) directly due to osseointegration-related complications, and/or (3) due to preexisting health problems exacerbated by osseointegration.

Demographic data were summarized with descriptive statistics. Means (SDs) were compared using an unpaired, 2-tailed *t* test; frequencies were compared using the Fisher exact test. To evaluate variables potentially associated with mortality, a regression analysis was performed considering the binary outcome (alive or deceased) in consideration of multiple variables. Categorical variables included sex, level of amputation (femur or tibia), laterality, amputation etiology (trauma, vascular, cancer, deformity, infection, or other), and whether patients ever had surgery to manage postosseointegration infection. Continuous variables included interval in years from amputation to osseointegration, age at osseointegration, and weight.

### Statistical Analysis

Annual mortality risk was estimated by performing Kaplan-Meier survival analysis. Log-rank analyses compared the risk of categorical variables; Cox proportional hazards regression modeling evaluated the risk of continuous variables. Statistical analyses and modeling were performed with RStudio: Integrated Development for R, version 4.2.1 (R Program for Statistical Computing) using the survival and survminer packages. Bonferroni or other correction was not performed given the sample sizes and desire to avoid a type II error. All significance was defined as 2-sided *P* ≤ .05.

## Results

A total of 485 patients were included in the analysis (345 men [71.1%] and 140 women [28.9%]), with a mean (SD) age at osseointegration of 49.1 (14.6) years among living patients or 61.2 (12.4) years among those who died. The Table presents the patient demographic characteristics and categorizations for alive and deceased patients. Nineteen patients (3.9%) died a mean (SD) of 2.2 (1.7) years (range, 58 days to 5 years) after osseointegration. Two of the 19 deaths (10.5%; 2 of 485 patients overall [0.4%]) were related to osseointegration—both of which were due to infectious complications, and 1 of which (0.2%) was coclassified as a preexisting health probem that was exacerbated by the procedure (myocardial infarction after subsequent surgery to manage infection). No deaths occurred after periprosthetic fracture or intraoperatively or were associated with inpatient recuperation or short-term perioperative complications after index osseointegration, cardiopulmonary or circulatory complications (eg, fat embolism or respiratory distress, pulmonary embolism), or operation-associated infections (eg, urinary, respiratory, or line-associated infection). The remaining 17 patient deaths were considered unrelated to osseointegration. The most common cause of death consisted of cardiac issues that occurred after and were unrelated to osseointegration. eTable 1 in the Supplement presents the follow-up duration of living and deceased patients.

**Table.** Patient Characteristics by Survival

Characteristic	Patient group^a^		*P* value^b^
	Alive (n = 466)	Deceased (n = 19)	
Age at surgery, mean (SD) [range], y	49.1 (14.6) [16-85]	61.2 (12.4) [42-75]	.002
Sex			
Men	328 (70.4)	17 (89.5)	.12
Women	138 (29.6)	2 (10.5)	
Time from amputation to osseointegration, mean (SD) [range], y	11.0 (13.9) [0-66]	5.8 (9.4) [0-40]	.03
Weight, mean (SD) kg	84.7 (20.8)	91.2 (18.5)	.18
Left side	183 (39.3)	8 (42.1)	.81
Femur level	320 (68.7)	11 (57.9)	.32
Time from TOPA to death, mean (SD) [range], y	NA	2.2 (1.7) [0.2-5.1]	NA
Etiology of amputation			
Trauma	311 (66.7)	7 (36.8)	.01
Infection	52 (11.2)	6 (31.6)	.02
Cancer	42 (9.0)	0	.39
Vascular	28 (6.0)	4 (21.1)	.03
Deformity	13 (2.8)	1 (5.3)	.43
Other	20 (4.3)	1 (5.3)	.58
Cause of death^c^			
0 to <90 d			
Suicide	NA	1 (5.3)	NA
90 d to <1 y			
Cancer	NA	3 (15.8)	NA
Suicide	NA	1 (5.3)	NA
Trauma	NA	1 (5.3)	NA
Pulmonary issues	NA	1 (5.3)	NA
1 to <2 y			
Cardiac issues	NA	3 (15.8)	NA
Cancer	NA	1 (5.3)	NA
Unknown	NA	1 (5.3)	NA
2-5 y			
Cardiac issues	NA	2 (10.5)	NA
Pulmonary issues	NA	2 (10.5)	NA
Osseointegration-related infection	NA	1 (5.3)	NA
Trauma	NA	1 (5.3)	NA
>5 y			
Osseointegration-related infection	NA	1 (5.3)	NA
Suicide	NA	1 (5.3)	NA

Abbreviations: NA, not applicable; TOPA, transcutaneous osseointegration post amputation.

^a^ Unless indicated otherwise, data are expressed as No. (%) of patients.

^b^ Continuous data were compared using an unpaired, 2-tailed *t* test; categorical data were compared using the Fisher exact test.

^c^ Causes of death were categorized as (1) unrelated to osseointegration, (2) directly due to osseointegration-related complications, and/or (3) due to preexisting health problems exacerbated by osseointegration. Although 19 patients died, the categories sum to 20 instead of 19 because patient 5 died from both category 2 and 3 reasons.

eTable 2 in the Supplement presents the results of the binary logistic regression modeling. Significant risk was identified for patients whose initial amputation was performed to manage infection (simple regression odds ratio [OR], 3.87 [95% CI, 1.31-11.40; *P* = .01]; multiple regression OR, 5.95 [95% CI, 1.78-19.89; *P* = .004]) and vascular causes (simple regression OR, 4.73 [95% CI, 1.35-16.56; *P* = .01]).

**Figure 2.**
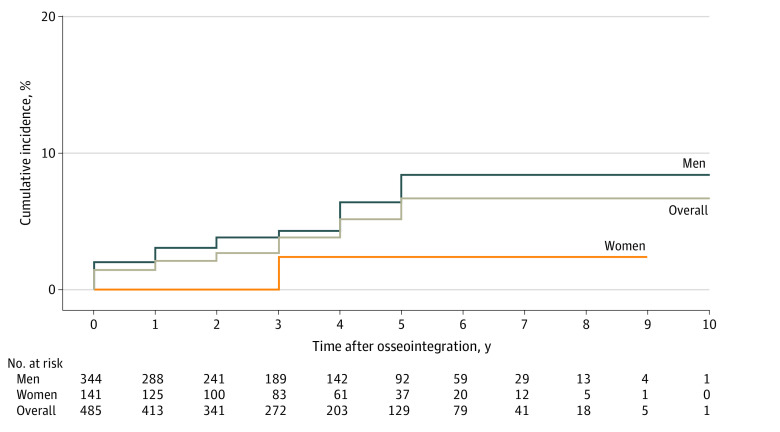
Kaplan-Meier Survival Analysis of the Entire Cohort and Stratified by Sex The graph’s vertical axis (cumulative incidence of mortality) is truncated to improve the visibility of the data. The log-rank comparison of male and female survivorship is presented (*P* = .05).

**Figure 3.**
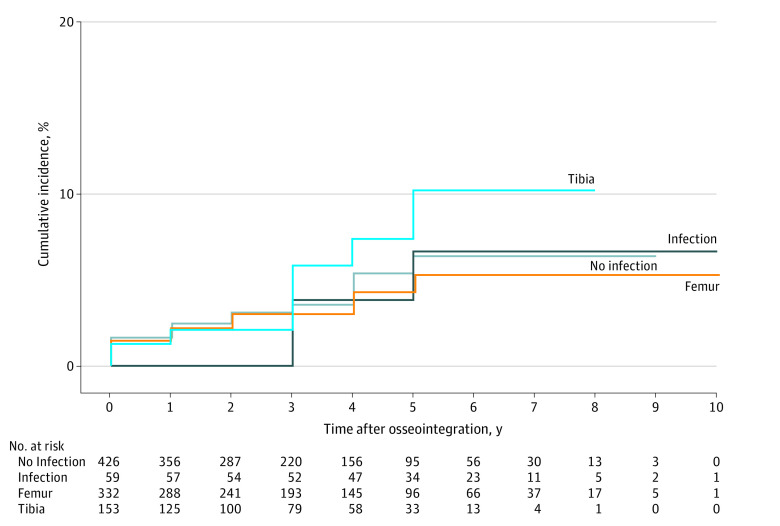
Kaplan-Meier Survival Analysis of Cohorts Stratified by Infection vs No Infection and Femur vs Tibia The graph’s vertical axis (cumulative incidence of mortality) is truncated to improve the visibility of the data. For infection vs no infection, log-rank *P* = .82; for femur vs tibia, log-rank *P* = .31.

**Figure 4.**
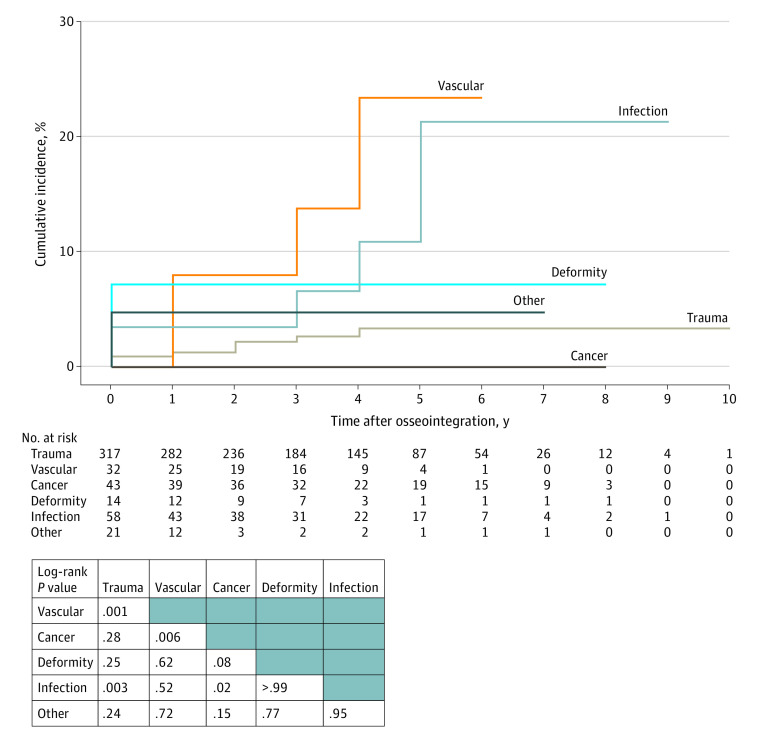
Kaplan-Meier Survival Analysis of the 6 Causes of Amputation That Were Evaluated The graph’s vertical axis (cumulative incidence of mortality) is truncated to improve the visibility of the data. The log-rank comparisons are presented for each pairwise test.

The survival analysis was performed using Kaplan-Meier survival modeling and presented as cumulative incidence. Figure 2 presents the incidence of mortality, both overall and dichotomized by sex. Male sex did not meet the threshold for significance as a risk factor based on log-rank comparison (hazard ratio [HR], 0.26 [95% CI, 0.06-1.13]; *P* = .05). Figure 3 presents the survival analysis and log-rank tests for level of amputation (femur vs tibia) (HR, 0.87 [95% CI, 0.25-1.98]; *P* = .82) and whether there was increased risk of postosseointegration mortality among the 59 patients who had operative intervention to manage postosseointegration infection (HR, 1.58 [95% CI, 0.65-3.87]; *P* = .31); neither were significant risks. Figure 4 presents survival based on amputation etiology. The following log-rank pairwise comparisons of estimated survival risk reached the threshold for significant difference: vascular vs trauma (HR, 5.95 [95% CI, 1.79-19.80]; *P* = .001), infection vs trauma (HR, 1.45 [95% CI, 1.11-1.89]; *P* = .003), cancer vs vascular (HR, 0.00 [95% CI, 0.00-infinity]; *P* = .006), and infection vs cancer (HR, 2.41 × 10^4^ [95% CI, 0.00-infinity]; *P* = .02). The Cox proportional hazards regression models evaluating the influence of continuous variables on survival is shown in eTable 3 in the Supplement. Increasing age at TOPA was a significant risk for mortality; each additional year of age conferred a hazard ratio of 1.06 (95% CI, 1.02-1.09; *P* = .002). Patient weight and the interval from amputation to osseointegration were not significant risks.

## Discussion

The most important finding of this cohort study is that our data suggest that mortality due to osseointegration-related complications after TOPA is rare. Only 2 of 485 patients (0.4%) died of osseointegration-related complications, and only 19 (3.9%) died of any cause during follow-up after osseointegration. Regression modeling and Kaplan-Meier survival analysis with log-rank pairwise comparison revealed that patients with amputations performed to manage vascular problems or infection have increased mortality vs those whose amputations were performed for other reasons, significantly greater than patients who underwent amputation for oncologic and traumatic causes. As might be expected, increasing patient age was a risk factor for mortality, but not owing to osseointegration-related issues. Because this study is the first, to our knowledge, to focus on postosseointegration mortality, there is no direct literature providing any context. Therefore, this discussion regarding mortality among patients who underwent osseointegration focuses on 3 topics. First, we contextualize this cohort’s mortality rate and causes of death vs other TOPA cohorts, patients with lower extremity amputations in general, and other populations of patients who have undergone major reconstructive orthopedic surgery. Second, we contextualize how osseointegration-related infection risk for mortality compares with infection risk in other major orthopedic reconstruction cohorts. Finally, the issue of suicide among patients who have undergone amputation is briefly addressed, both generally and with respect to osseointegration.

Although no prior research directly investigates postosseointegration mortality, Hagberg et al^10^ reported 3 deaths in 111 patients (2.7%) after as much as 15 years of follow-up. Reetz et al^11^ reported 1 death among 39 patients (2.6%). Neither group reported the causes of death, stating only that they were unrelated to TOPA. Örgel et al^12^ reported 1 case of intraoperative mortality due to pulmonary embolism, but no articles from that group could be identified that mention postoperative mortality within their cohort of more than 140 patients.^13^ Most recently, Reif et al^14^ reported no deaths among 31 patients. With 485 patients with transfemoral and transtibial osseointegration in total, our cohort represents 170% of the cumulative patient total in the 4 cited publications combined. Our all-cause mortality rate was 3.9%, and the TOPA-associated mortality rate was 0.4%.

The field of osseointegration is relatively new and the surgery is performed by very few groups, justifying the paucity of investigations regarding mortality. However, lower extremity amputation is not new, yet mortality remains understudied, with the relative exception of patients with diabetes- and vascular disease–related amputations. A recent systematic review of long-term mortality after nontraumatic lower extremity amputation, which was overwhelmingly representative of patients with vasculopathy and diabetes,^15^ identified the mortality rate at 1 year as 33.7%, at 2 years as 51.5%, and at 5 years as 64.4%. Mortality data on other populations of individuals who have undergone amputation is much more limited; for example, 1 multicenter trial^16^ reported no mortality through 2 years among 149 patients. Our cohort was decidedly heterogeneous regarding indications for primary amputation and timing of osseointegration in relation to the original amputation, so unfortunately this study was not designed to directly compare against studies following a single indication for amputation.

Mortality after total hip (THA) and knee (TKA) arthroplasty has been studied more frequently. Although total joint replacement is substantially different from osseointegration, we consider comparison with these patient groups purposeful because both osseointegration and joint replacement are performed to improve patient mobility and quality of life, usually electively, and feature relatively large, permanent implants in extremities. Studies investigating standardized mortality ratios identify that historically, the 90-day period after THA or TKA was associated with worse standardized mortality ratios.^17^ However, during the last 20 years, patients who underwent THA and TKA tended to have similar or better standardized mortality ratios vs the average population,^18^ with the benefit persisting for approximately 1 decade after surgery.^19^ The acute improvement in survival is attributed to continuously enhanced anesthesia and postoperative protocols, with the longer-term survival attributed to patient selection and preoperative medical optimization.^20^ Other studies^21,22^ have reported 90-day THA and TKA mortality as ranging from 0.4% to 0.6%; in our cohort, TOPA-related mortality was 0.2%. A systematic review^23^ identified cardiac complications as the most common cause of death (0.1%-0.3%), followed by pulmonary emboli (0.1%).^22^ Long-term studies^19,24^ identify cardiac, vascular, cancer, and cerebrovascular events as the most frequent causes of death. Our cohort’s leading causes of death were cancer, pulmonary issues, and cardiac issues, consistent with those studies, but suicide was a leading cause of death in our cohort that was not identified in the THA and TKA studies. Beyond joint replacement orthopedic surgery, mortality after noncervical spine surgery has an estimated short-term mortality of 0.4% to 0.7%,^25,26^ and even total shoulder^27^ and total ankle arthroplasty^28^ have nonnegligible short-term mortality rates. These studies emphasize how and why understanding the rates and causes of mortality after surgery is important to properly counsel patients.

Infection remains the paramount concern for TOPA, but its association with mortality has not been previously evaluated. Of our 485 patients with TOPA of the lower extremity, 59 (12.2%) required at least 1 subsequent operative intervention for infection, and both patients in this study who died of osseointegration-related complications had infection originating from the stoma site. Preventing stoma infection is a complex issue not only for transcutaneous extremity osseointegration but for any situation involving long-term transcutaneous bone implants. The most common such situation, and the most studied, is for external fixation. External fixator pin site infection is estimated to occur in 4% of patients who have undergone nonacute trauma deformity correction.^29^ Approximately 28% of patients with external fixation applied as part of acute trauma care develop infection,^30^ and 2.5% of pins become clinically loose, necessitating their removal.^31^ Repetitive microtrauma to the pin site leading to local inflammation and bacterial ingress is believed to increase the susceptibility to infection.^29^ Because external fixation is temporary instead of permanent, patients typically can be managed with suppressive antibiotics until the correction is achieved and the fixator removed, when the skin can close and end the cycle of colonization and infection.^29^ Patients rarely have long-term infection sequelae or death after temporary external fixation. This outcome is different from prosthetic joint infection. A 2019 review of 19 studies of prosthetic joint infection^32^ estimated the annual mortality risk after prosthetic joint infection managed with 2-stage revision to be 4.2%, or a 5-year risk of 21.1%, compared with an age-matched population mortality of 1.58%. This survival rate is reportedly worse than rates for the 4 most common cancers: testicular, Hodgkin lymphoma, melanoma, and breast cancer.^33^ Of our 59 patients who had surgery to manage postosseointegration infection, 2 died (3.4%) at 3 years and more than 5 years after osseointegration.

Three patients in this cohort committed suicide, the third-largest cause of mortality for our patients. Although these suicides seem unlikely to be directly related to TOPA, they are arguably preventable and are certainly a devastating event for the patient’s family and for the physician. Suicide in relation to osseointegration has not been previously investigated, but individuals who have undergone amputation have long been recognized as a population at risk for mental health issues, including suicide. Pierce et al^34^ highlighted several associated risks among individuals who have undergone amputation: unemployment, substance abuse, additional surgical procedures, chronic pain, and mobility problems associated with prosthesis sockets. The rate of suicidal ideation among these patients varies and depends on many factors, but it has been estimated to be at 15% to 30%.^35,36^ A full discussion of mental health and suicidal ideation among this population far exceeds the scope of this article, but some points are common and noteworthy. Individuals who have undergone amputation rely on caregivers, particularly in the immediate postoperative and early rehabilitation periods. The spouse or other family member is often the caretaker, and this arrangement can stress relationships and lead to depressive symptoms.^37^ Depressive symptoms may be the most reliable factor associated with suicidal ideation,^38^ and improved independence^35^ and rehabilitation appear to be protective against depressive symptoms and suicidality.^39^ Given that most patients improve their quality of life and mobility after TOPA,^2^ and the recent investigation by Reif et al^14^ also identifies improved mental health, it seems reasonable to offer this reconstruction to appropriately selected patients.

Our preoperative evaluation includes several components that have been identified as protective against suicide risk: an integrated care team including a physiatrist, physiotherapist, prosthetist, and internist; separate formal consultation with a clinical psychologist; and formal inquiries regarding patients’ mental health, with both preoperative and postoperative questionnaires.^35^ The 3 patients who committed suicide had preexisting mental health conditions (bipolar disorder, posttraumatic stress disorder, and/or depression) but were assessed as stable and well supported before surgery. Why, then, might they have committed suicide? Notably, these patients also had phantom pain and neuroma pain that was incompletely improved after TOPA. It is possible that the lack of this specific pain relief could have been a factor behind their suicide. There is optimism that providing targeted muscle reinnervation (TMR) and regenerative peripheral nerve interface (RPNI) surgery, techniques recently identified to improve nerve-related pain among patients who have undergone amputation, may provide better pain improvement for all patients and possibly reduce this specific risk of suicide in those who are at risk.^40^

### Strengths and Limitations

The main strengths of this study include the relatively large cohort size (485 patients) and the high rate of recent follow-up (93.8% contact within the prior 2 years). Furthermore, the cause of death was known for all but 1 patient. The influence of several major factors (sex, age, weight, and time since amputation) were assessed, and the rates of mortality were compared for several relevant variables (age, sex, level of amputation, postoperative infection, and amputation etiology).

The major limitations of this study are those inherent in any longitudinal cohort study, including selection bias and reporting bias. In addition, a greater rate of procedures was performed each year, so longer-term follow-up has lower relative representation. In addition, the mortality rates may be influenced by the relatively strict patient selection criteria used in our group’s early experience with osseointegration: young and healthy patients were generally considered more suitable candidates for osseointegration initially. The indications were gradually expanded as procedural familiarity and generally positive outcomes guided counseling for patients considered to be potentially at greater risk. Generalizability of the results is likely most contingent on the health of patients or cohorts.

## Conclusions

The findings of this cohort study suggest that patients who have had osseointegration rarely die of complications associated with the procedure. Infection remains the most common complication associated with osseointegration and is the only osseointegration-related cause of death in our cohort. As with any population of individuals who have undergone amputation, clinicians should routinely inquire about mental health for general purposes but also specifically to help prevent suicide. In general, most patients who have had osseointegration will most likely survive until unrelated medical or accidental events are responsible for their death.

## Notes

Supplement. **eTable 1.** Follow-up Duration for Patients **eTable 2.** Binary Logistic Regression Analysis Results: Likelihood of Mortality **eTable 3.** Cox Proportional Hazards Model of Mortality Risk
