# Supplementary material for: Association Between Osseointegration of Lower Extremity Amputation and Mortality Among Adults
Source: JAMA Netw Open. 2022 Oct 13;5(10):e2235074. doi: 10.1001/jamanetworkopen.2022.35074 (PMC9561949; doi:10.1001/jamanetworkopen.2022.35074)
Supplement: Supplement. — eTable 1. Follow-up Duration for Patients eTable 2. Binary Logistic Regression Analysis Results: Likelihood of Mortality eTable 3. Cox Proportional Hazards Model of Mortality Risk [file jamanetwopen-e2235074-s001.pdf]

Supplementary Online Content

Hoellwarth JS, Tetsworth K, Oomatia A, Akhtar MA, Xu H, Al Muderis M. Association between osseointegration of lower extremity amputation and mortality among adults. *JAMA Netw Open*. 2022;5(10):e2235074. doi:10.1001/jamanetworkopen.2022.35074

eTable 1. Follow-up Duration for Patients
eTable 2. Binary Logistic Regression Analysis Results: Likelihood of Mortality
eTable 3. Cox Proportional Hazards Model of Mortality Risk

This supplementary material has been provided by the authors to give readers additional information about their work.

© 2022 Hoellwarth JS et al. *JAMA Network Open*.

eTable 1. Follow-up Duration for Patients

Years since Osseointegration	Years Followed	Count	Deceased
10	10	3	
	9	2	
9	9	11	
	8	4	
	7	2	
	3	1	1
	1	1	1
8	8	14	
	7	3	
	6	1	
	4	1	
	3	1	
7	7	21	
	6	8	
	5	5	
	4	1	
	3	1	
	2	1	
	1	1	1
6	6	35	
	5	15	3
	4	5	
	3	2	1
	2	2	
	1	2	
	0	1	
5	5	49	1
	4	10	2
	3	4	
	2	8	
	1	4	
	0	4	1
4	4	53	
	3	10	
	2	10	

© 2022 Hoellwarth JS et al. *JAMA Network Open*.

	1	3	
	0	5	
3	3	51	1
	2	10	1
	1	5	1
	0	1	
2	2	40	2
	1	11	1
	0	4	1
1	1	39	1
	0	6	
0	0	14	

© 2022 Hoellwarth JS et al. *JAMA Network Open*.

eTable 2. Binary Logistic Regression Analysis Results: Likelihood of Mortality

Category	**Simple (Single Category)** Regression			**Multiple (All Categories)** Regression		
	p-Value	Odds Ratio	95% conf. interval	p-Value	Odds Ratio	95% conf. interval
Gender Male	(control)	(control)	(control)	(control)	(control)	(control)
Female	0.126	.35	.09 - 1.35	.168	.415	.11 - 1.46
Level Femur	(control)	(control)	(control)	(control)	(control)	(control)
Tibia	.358	1.55	.61 - 3.93	.085	2.39	.89 - 6.42
Side Left	(control)	(control)	(control)	(control)	(control)	(control)
Right	.624	1.28	.48 - 3.43	.332	1.65	.60 - 4.53
Bilateral	.916	.91	.16 - 5.12	.94	.93	.13 - 6.71
Etiology Trauma	(control)	(control)	(control)	(control)	(control)	(control)
Infection	.014	3.87	1.31 - 11.40	.004	5.95	1.78 - 19.89
Cancer	.587	.47	.03 - 7.01	.687	.51	.02 - 13.65
Vascular	.015	4.73	1.35 - 16.56	.069	3.99	.90 - 17.75
Deformity	.800	1.43	.09 - 22.51	.822	1.41	.07 - 28.55
Other	.934	1.11	.09 - 14.14	.560	1.94	.21 - 18.14
Postoperative Debridement No	(control)	(control)	(control)	(control)	(control)	(control)
Yes	.656	1.34	.37 - 4.94	.344	1.79	.54 - 6.01
Years Amputation to Implantation	.418	.98	.94 - 1.02	.414	.98	.93 - 1.03
Age (years)	.302	1.02	.99 - 1.04	.34	1.02	.98 - 1.05
Weight (kg)	.957	1.00	.98 - 1.02	.74	1.00	.97 - 1.02

© 2022 Hoellwarth JS et al. *JAMA Network Open*.

eTable 3. Cox Proportional Hazards Model of Mortality Risk

Category	Individual Category Cox			All Categories Cox		
	p-Value	Hazard Ratio	95% conf. interval	p-Value	Hazard Ratio	95% conf. interval
Years Amputation to Osseointegration	.073	.95	.90 - 1.00	.055	.95	.90 - 1.00
Age	.002	1.06	1.02 - 1.09	.001	1.02	1.02 - 1.09
Weight	.11	1.02	1.00 - 1.04	.187	.99	.99 - 1.04

© 2022 Hoellwarth JS et al. *JAMA Network Open*.
